# p53 Cooperates with Sp1 to Regulate Breed-Dependent Expression of Glucocorticoid Receptor in the Liver of Preweaning Piglets

**DOI:** 10.1371/journal.pone.0070494

**Published:** 2013-08-07

**Authors:** Huafeng Zou, Zheng Jiang, Runsheng Li, Yimin Jia, Xiaojing Yang, Yingdong Ni, Ruqian Zhao

**Affiliations:** Key Laboratory of Animal Physiology and Biochemistry, Ministry of Agriculture, Nanjing Agricultural University, Nanjing, P. R. China; University of Saarland Medical School, Germany

## Abstract

Previous studies indicate that Chinese indigenous pig breeds demonstrate distinct pattern of glucocorticoid receptor (GR) expression, which is associated with their unique growth and metabolic phenotypes. Here we sought to unravel the transcriptional mechanisms underlying the breed-specific hepatic GR expression in preweaning Chinese Erhualian (EHL) and Western Large White (LW) piglets. Total GR mRNA and the predominant GR mRNA variant 1–9/10 were expressed significantly higher in EHL compared with LW piglets (*P*<0.01), which was associated with more enriched histone H3 acetylation on 1–9/10 promoter (*P*<0.05). Nuclear content of transcription factor specificity protein 1 (Sp1) was significantly lower in EHL piglets, yet its binding to GR 1–9/10 promoter was significantly higher in EHL piglets, as revealed by chromatin immunoprecipitation assays. Although p53 binding to GR promoter 1–9/10 did not differ between breeds, expression of p53 mRNA and protein, as well as its binding to Sp1, were significantly higher in EHL piglets. Moreover, p53 activator doxorubicin significantly enhanced GR 1–9/10 promoter activity in HepG2 cells at 100 nM, which was associated with significantly higher protein content of p53 and GR. Sp1 inhibitor, mithramycin A, significantly inhibited (*P*<0.05) the basal activity of GR promoter 1–9/10 and completely blocked doxorubicin -induced activation of GR promoter 1–9/10. These data indicate that higher hepatic GR expression in EHL piglets attributes mainly to the enhanced transcription of GR promoter 1–9/10, which is achieved from breed-specific interaction of p53 and Sp1 on porcine GR 1–9/10 promoter.

## Introduction

Glucocorticoid receptor (GR) mediates most functions of glucocorticoids and hence plays indispensable roles in almost all aspects of life including growth and differentiation, energy homeostasis and obesity, as well as immunity and stress responses [1], [2]. Although studies on domestic animals are relatively scarce compared to those on human and rodents, accumulating evidences suggest important roles of GR in livestock traits of agricultural interests, including carcass and meat quality, stress sensitivity and immunity, as well as animal health and welfare [3], [4]. Previous studies indicate that Chinese indigenous pig breeds demonstrate distinct pattern of GR expression in hippocampus [5], liver [6] and muscle [7] compared with western pig breeds, which is associated with the breed-specific characteristics in stress coping style, hepatic gluconeogenesis, and intramuscular fat deposition, respectively. Therefore, GR is regarded as a regulatory target not only in human medical research but also in animal science aiming to improve livestock performances through manipulating GR expression.

Expression of GR gene is controlled in a temporal, spatial and tissue-specific manner by sophisticated mechanisms at different levels, including chromatin condensation, transcription initiation, alternative RNA splicing, mRNA stability and others. Among all these regulatory mechanisms, the progression of transcriptional regulation is known to play a major role [8]–[10]. Multiple alternative first exons are located in the proximal and distal promoter region of GR gene, and the tissue-specific usage of alternative first exons leads to different GR mRNA transcripts, which provides a mechanism for the local fine-tuning of GR mRNA levels [11], [12]. Apart from the tissue-specific pattern, GR expression also demonstrates developmental changes and strain or breed disparities [13]–[15], adding further complexity to the transcriptional regulation of GR expression. We reported recently that 5′-untranslated GR exon 1 mRNA variants 1–4 and 1–5, as well as the total GR mRNA are expressed in a breed-dependent manner in the liver of newborn piglets, Large White (LW) showing significantly higher expression compared with Erhualian (EHL), a Chinese indigenous breed [17]. This breed difference contradicts with the previous findings that Chinese pigs usually express higher GR compared to Western pig breeds in hippocampus [1], liver [2] and muscle [3] at later ages after weaning. It appears that the breed-specific GR expression in the pig is age-dependent. The questions arise whether hepatic GR expression is regulated differently at weaning compared to the newborn stage, and what mechanisms are involved in the breed disparity of GR transcriptional regulation in the pig liver before weaning.

A wide variety of transcription factors have been identified to bind to the cis-acting elements on GR promoter to regulate GR transcription, among which are specificity protein 1 (Sp1) [16], YinYang 1 [17], cAMP response element binding protein [18] and nerve growth factor-inducible protein A [19]. Sp1, a ubiquitous transcription factor, plays an important role in the constitutive transcription of GR in most tissues [16], [20]. Sp1 can bind to GC-rich sequences or GC box on gene promoters to regulate gene transcription. Moreover, a number of other transcription factors, including p53, GATA and YinYang 1, are reported to interact with Sp1 to fine-tune its function in a tissue-specific manner [21]. p53, a tumor suppressor protein, functions as a transcription factor to regulate cell cycle progress and apoptosis by directly binding to p53-binding consensus sequences on the promoter of its target genes [22], or by interacting with other transcription factors to regulate the expression of genes lacking a p53 response element. A number of studies have implicated the correlation between p53 and GR [23], [24] or GR target genes [25]. However, little is known whether and how p53 participates in the transcriptional regulation of GR expression.

To understand the possible involvement of Sp1 and p53 in the transcriptional regulation of breed-specific hepatic GR expression, we use preweaning EHL and LW piglets as model. First, we compared the hepatic expression of total and proximal first exon transcript variants of GR mRNA between the two breeds of piglets; Second, we analyzed the correlation of Sp1 and p53 with GR expression; and third, we investigated the role of Sp1 and p53 in the regulation of differentially transcribed GR first exon mRNA variant in the liver of preweaning piglets. In addition, p53 activator doxorubicin (Dox) and Sp1 inhibitor mithramycin A (Mit) were used to confirm the involvement of p53 and Sp1 in the transactivation of GR 1–9/10 promoter in HepG2 cells in vitro.

## Results

### Body weight, liver weight and serum cortisol level differ between breeds

As shown in Table 1, EHL piglets were only about one-half of the body weight of LW piglets on 25 d of age (*P*<0.01). Accordingly, liver weight was also significantly lower in EHL piglets (*P*<0.01), yet the liver index (liver weight relative to body weight) was significantly higher. Serum cortisol concentration was 3-folds higher in EHL piglets compared with LW piglets (*P*<0.01).

**Table 1:** Body weight, liver weight and serum cortisol concentration in preweaning piglets.

Parameters	LW	EHL	*P* value
Body weight (kg)	8.10±0.34	4.08±0.25	*P*<0.05
Liver weight (g)	178.74±15.40	119.84±6.72	*P*<0.05
Relative liver weight (g/kg)	21.92±1.15	29.51±1.10	*P*<0.01
Serum cortisol (ng/mL)	32.64±9.47	101.42±8.79	*P*<0.01

Values are mean ± SEM, n = 5.

### Expression of total GR mRNA and GR exon 1–9/10 mRNA variant is breed-dependent

**Figure 1:**
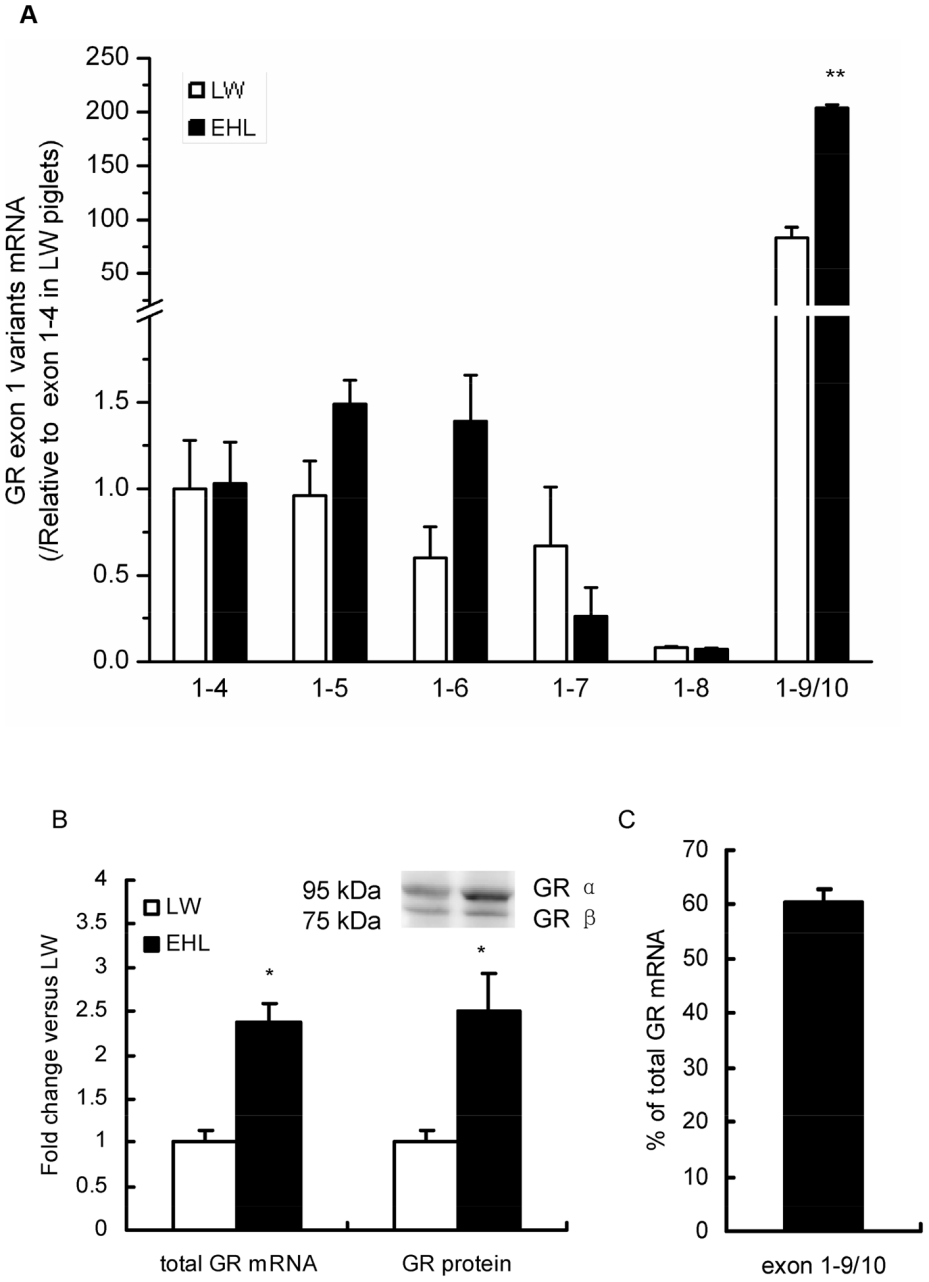
Expression of GR first exon mRNA variants, total GR mRNA and protein in the liver of preweaning piglets. (A) Expression of GR first variants expression in liver, 18S are used as the reference gene; (B) Expression of total GR mRNA and protein in liver; (C) Percentage of exon 1–9/10 mRNA relative to total GR mRNA in liver of piglets. Values are mean ± SEM, n = 5. *, *P*<0.05, **, *P*<0.01, compared with LW piglets.

Among all the proximal GR exon 1 mRNA variants detected, only GR exon 1–9/10 mRNA variant was differentially expressed in the liver of preweaning LW and EHL piglets. Significantly higher abundance of GR 1–9/10 mRNA was detected in EHL piglets compared to LW (Figure 1A). Total GR mRNA and protein in the liver were significantly higher in EHL piglets (Figure 1B). The standard curve of GR exon 1–9/10 variant and total GR was generated as linear regression between Ct and log10 copy number of standard DNA (Figure S1). The absolute quantification of GR 1–9/10 and total GR mRNA revealed that GR 1–9/10 variant was predominantly expressed in porcine liver, which comprises approximately 60% of the total GR mRNA (Figure 1C). This finding is in line with a recently published paper showing that exon 1-9/10 is the predominant variant in porcine liver which accounts for about 65% of total GR mRNA [26].

### Nuclear protein content of Sp1 is lower but its binding to GR promoter 1–9/10 is higher in EHL piglets, which was associated with higher enrichment of histone H3 acetylation (H3Ac) modification

**Figure 2:**
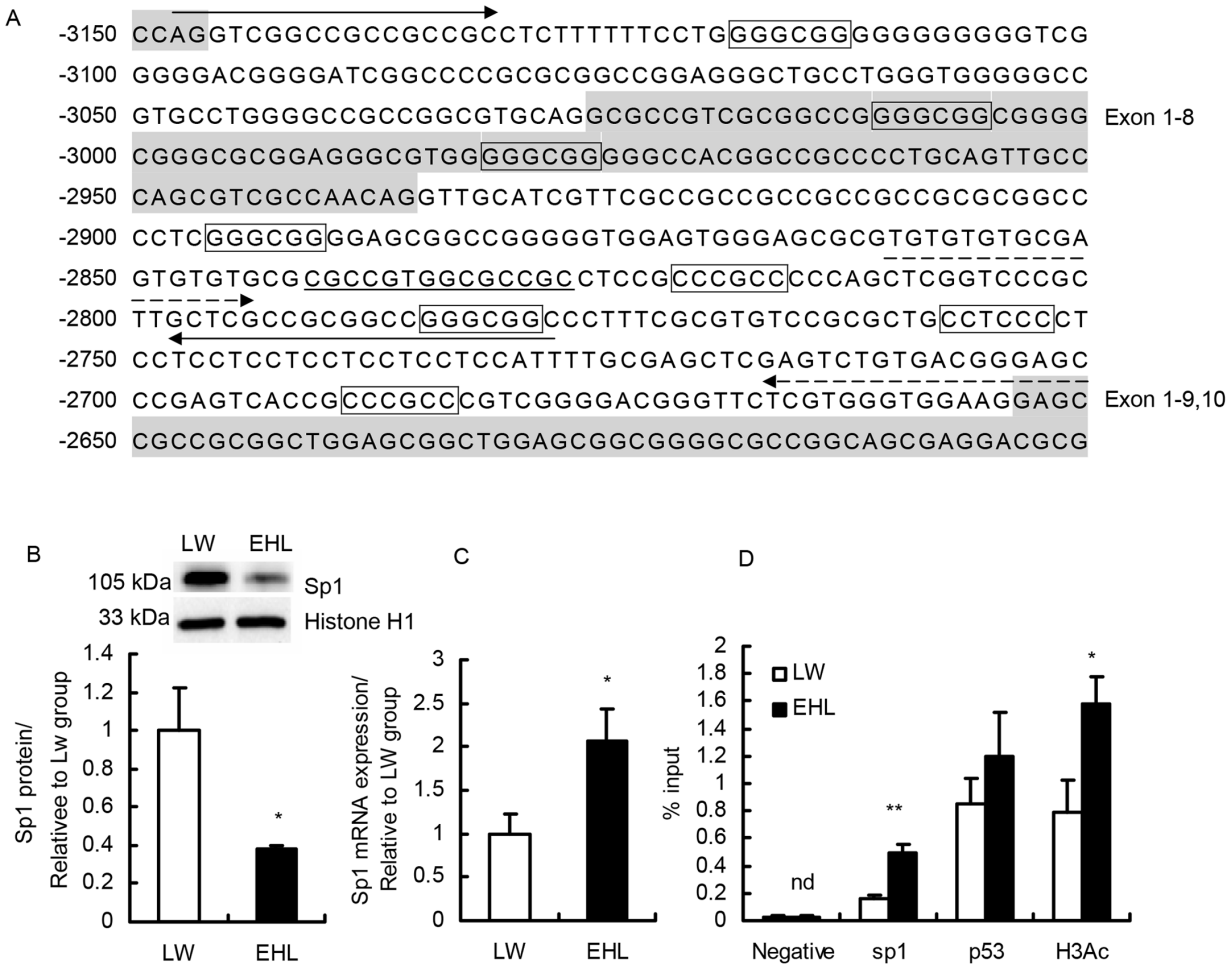
Protein and mRNA expression of Sp1 and ChIP analysis of porcine GR 1–9/10 promoter. (A) Sequence of porcine GR promoter 1–9/10 analyzed. Shaded sequences represent identified porcine alternative GR first exons (1–8 and 1–9/10). Primers for luciferase assay and ChIP assay are indicated by a solid arrow and a dotted arrow, respectively. The predicted Sp1 binding sites are marked in boxes, the underlined sequence is a sp1 binding site confirmed by EMSA [20]; (B) Nuclear content of Sp1 protein in liver of piglets, histone H1 was used a loading control; (C) Sp1 mRNA expression in liver of piglets, 18S was used as the reference gene; (D) ChIP analysis of Sp1, p53 binding and H3Ac enrichment on porcine GR promoter 1–9/10. The amount of precipitated DNA was calculated relative to the input, the ChIP results are presented as the ratio of the input DNA, nd means not detected. Values are mean ± SEM, n = 5. *, *P*<0.05, **, *P*<0.01, compared with LW piglets.

Three conserved Sp1 binding sites were predicted on GR promoter 1–9/10 (−2811/−2648) with both TESS (http://www.cbil.upenn.edu/tess) and MatInspector (http://www.cbrc.jp/research) programs (Figure 2A). Nuclear protein content of Sp1 was significantly lower in the liver of EHL pigs (*P*<0.05, Figure 2B), while Sp1 mRNA level was significantly higher (*P*<0.05, Figure 2C). Chromatin immunoprecipitation (ChIP) assay revealed that Sp1 binding to GR promoter 1–9/10 was significantly higher (*P*<0.01) in EHL piglets, which was associated with significantly more enriched (*P*<0.05) histone H3 acetylation on GR promoter 1–9/10. No breed difference was detected for p53 binding to GR promoter 1–9/10 (Figure 2D).

### p53 mRNA is positively correlated with GR mRNA and the interaction of p53 and Sp1 is breed-dependent

**Figure 3:**
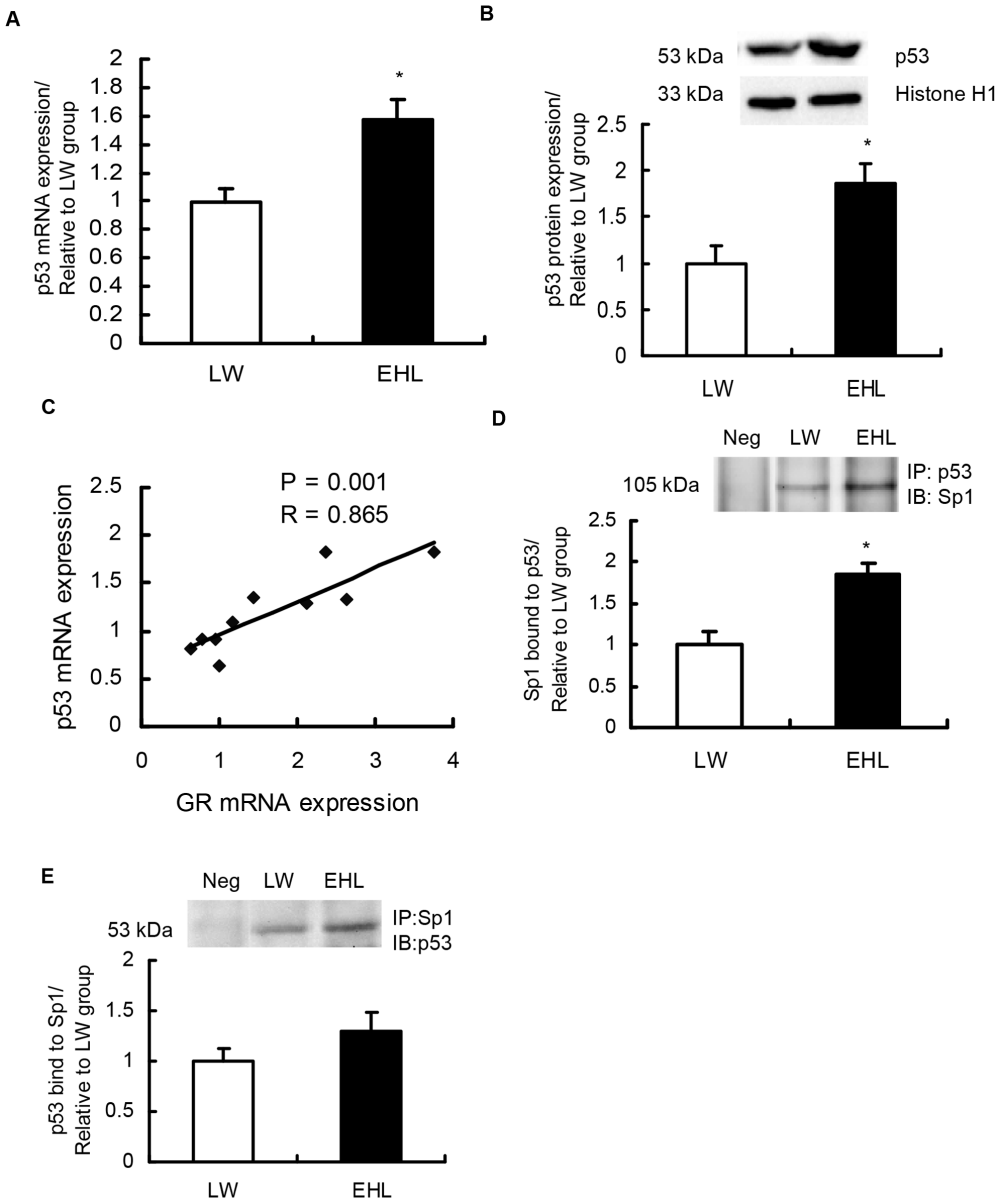
Expression of p53 and its associations with GR and Sp1 in the liver of preweaning piglets. (A) Expression of p53 mRNA in liver tissue, 18S was used as the reference gene; (B) Nuclear p53 protein content; Histone H1 was used as a loading control for Western Blot of p53, data were presented as the fold change relative to LW. (C) Correlation of p53 and GR mRNA levels; (D) and (E) Physical interaction of Sp1 and p53 was revealed by co-immunoprecipitation and Western blot. Lane 1 means negative control, lane 2 means LW, lane 3 mean EHL. Data were presented as the fold change relative to LW. Values are mean ± SEM, n = 4. *, *P*<0.05, compared with LW piglets.

p53 was expressed significantly higher in EHL piglets at the level of mRNA (Figure 3A) and nuclear protein (Figure 3B). A significant positive correlation (R = 0.865, *P*<0.01) was detected between GR mRNA and p53 mRNA in the liver of preweaning piglets (Figure 3C). Although p53 binding to GR 1–9/10 promoter did not differ between breeds (Figure 2D), co-immunoprecipitation analysis revealed the interaction of Sp1 and p53 in the nuclear protein extracts of piglet liver (Figure 3D, 3E). Interestingly, despite lower nuclear Sp1 content detected in EHL piglets, Sp1 that co-immunoprecipitated with p53 was significantly higher in EHL (Figure 3D), compared to LW piglets, implicating a role of p53 in the regulation of Sp1 action.

### p53 activator Dox significantly increases GR 1–9/10 promoter activity and endogenous GR expression in HepG2 cells

**Figure 4:**
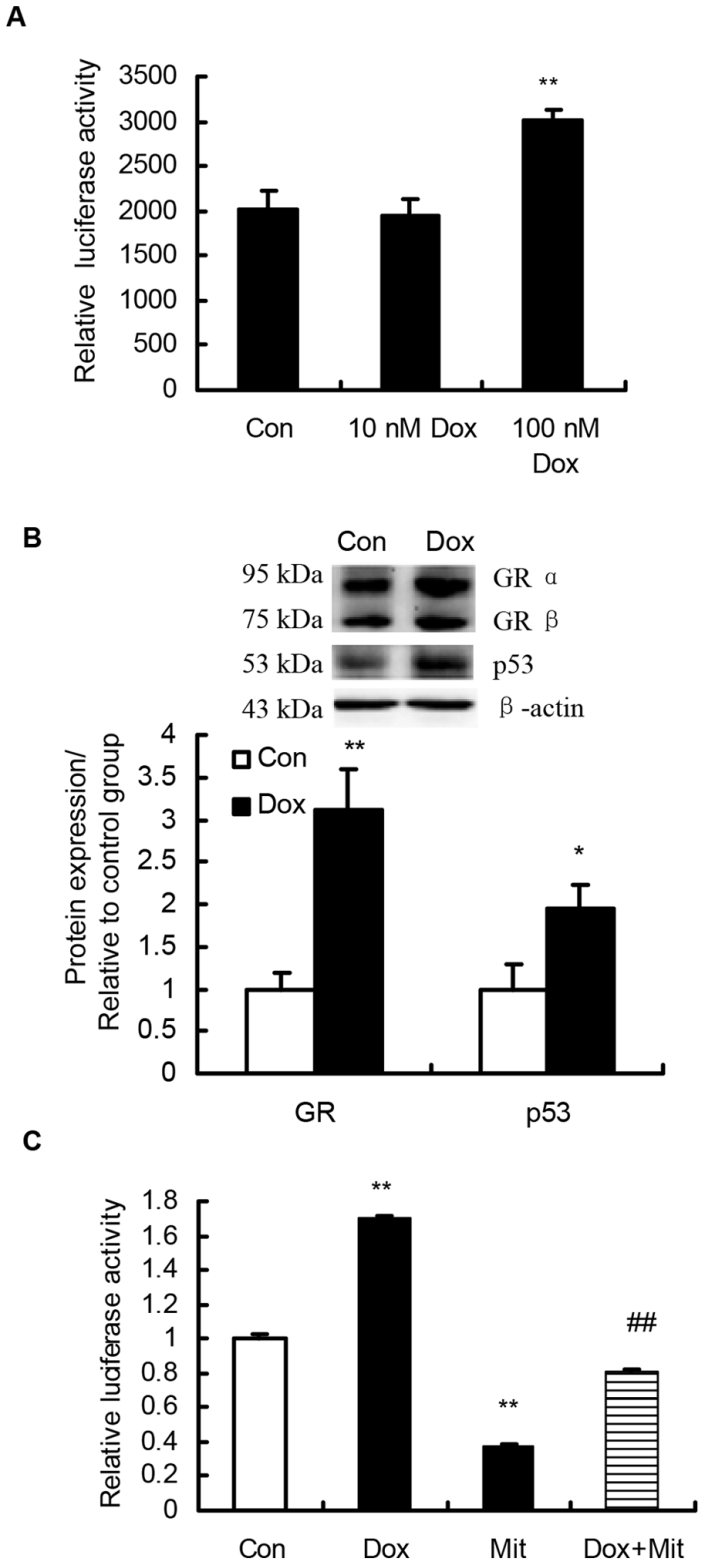
Effect of Dox and Mit on transcriptional activity of porcine GR 1–9/10 promoter in HepG2 cells. (A) Addition of Dox up-regulates GR promoter activity, the promoter sequence spans from –3149 to −2724 in relation to the translation start codon (ATG) of exon 2, which includes the intron prior to exon 1–9/10, as well as exon 1–8 (Figure 2A), luciferase activities are shown relative to the activity of the pGL3-basic vector, which was arbitrarily set to 1; (B) Mit inhibited Dox-mediated stimulation GR promoter 1–9/10-pGL3 activity. Luciferase activity in the cell lysate was normalized with Renilla luciferase activity of pRL-TK as an internal control, the activity for the promoter in the control group was arbitrarily set at 1, pGL3-basic plasmid was used for a negative control. ** *P*<0.01 compared with control. ##, *P*<0.01 compared with Dox group; (C) Protein content of GR and p53 in HepG2 cells treated with 100 nM Dox. β-actin was used as a loading control, p53 and GR protein contents are presented as fold change relative to control. Values are mean ± SEM, n = 4. * *P*<0.05, ** *P*<0.01, compared with control. Dox: Doxorubicin, Mit: Mithramycin A.

A luciferase report system for porcine GR 1–9/10 promoter (−3149, −2724) containing 8 Sp1 binding sites was generated as described in Materials and Methods (Figure 2A). Dox, at the concentration of 100 nM, but not 10 nM, significantly up-regulated GR 1–9/10 promoter activity (*P*<0.01) in transfected HepG2 cells (Figure 4A). The transactivation of reporter gene was associated with significantly increased (*P*<0.05) protein content of p53 and GR in the whole cell lysates of HepG2 cells (Figure 4B).

### Sp1 inhibitor Mit significantly inhibited the basal activity of GR promoter 1–9/10 and blocked Dox-induced activation of GR promoter 1–9/10

When HepG2 cells were treated with 100 nM of Sp1 inhibitor, Mit A, the basal activity of porcine GR promoter 1–9/10 was significantly decreased (*P*<0.01). Dox at 100 nM significantly increased promoter activity, while 100 nM of Mit completely blocked p53-mediated activation of porcine GR promoter 1–9/10 (Figure 4C).

## Discussion

The functional complexity of glucocorticoid is largely achieved by complex transcriptional regulation of GR, which involving multiple un-translated alternative first exons and their respective promoters [11]. We reported previously that in newborn piglets, GR exon 1–4 and 1–5 mRNA variants are expressed in breed-dependent manner in liver, showing higher abundance of GR exon 1–4, 1–5 and total GR mRNA in Large White piglets compared to Chinese EHL piglets [27]. To our surprise, this breed difference was reversed in the preweaning piglets. EHL piglets expressed higher abundance of total GR mRNA compared with LW piglets. Previous studies have shown that serum cortisol level is reversely correlated to GR content in liver [2], [3]. However, we found that although serum cortisol was consistently higher in both newborn and preweaning EHL piglets [27], GR expression showed reversed breed differences in newborn and preweaning stages. Therefore, the relationship between serum cortisol level and hepatic GR content is complex; many factors are involved in the determination of such ligand-receptor interaction. Furthermore, the differentially expressed GR mRNA variants also changed from GR exon 1–4 and 1–5 at birth to GR exon 1–9/10 before weaning. This developmental transit in hepatic GR transcription indicates different regulatory mechanisms, and also implicates different functionality of GR at different physiological stages in the pig. It has been shown that in fetal and neonatal liver, genes responsible for DNA replication are highly activated, while most of the metabolic genes are under expressed [28]. It is likely, therefore, that in developing fetus and newborns GR mainly regulates organogenesis and tissue growth which involve cell proliferation and differentiation. This presumption is supported by lack of response in some glucocorticoid-inducible hepatic enzymes to glucocorticoid in fetal liver [29]. The higher hepatic GR expression in newborn LW piglets is associated with heavier birth weight and liver weight in our previous study [27], which is also in line with the growth-promoting role of GR in neonatal stage. After birth when the organogenesis is complete, the function of GR may shift predominantly to metabolic regulation. Chinese pig breeds demonstrate earlier sexual maturation and the hepatic metabolic pathways are also activated earlier compared to the western pig breeds [6]. In this study, higher GR expression was detected in the liver of EHL piglets before weaning, which may be associated, at least partly, with enhanced glucose and lipid metabolic activity.

Developmental pattern of GR expression in liver has been documented previously in rats and pigs [30]–[32]. However, to our knowledge, here we provide the first evidence that breed-dependent expression of GR mRNA variants changes at different developmental stages. It has been shown that GR expression is predominantly regulated at transcriptional level [8]–[10] and a number of transcription factors are involved in the developmental regulation of GR expression. It has been shown that transcription factor Sp1, the most important transcription factor of GR expression, is expressed in age-dependent pattern. A postnatal up-regulation was observed at the age of 35 d in mouse liver [33]. Therefore, we naturally chose Sp1 as the major candidate transcription factor in our further investigation into the mechanisms of GR transcriptional regulation.

Sp1 is a key transcription factor involved in the regulation of diverse genes that lack a TATA-box [34]. At least five Sp1 consensus binding sites are reported to locate in the promoter of human GR exon 1C, the human orthologue of porcine GR 1–9/10 [20], [35]. In porcine GR gene, 3 conserved Sp1 binding sites were predicted in the intron region immediately 5′ of exon 1–9/10. Sp1 protein content in liver nuclear extracts was significantly lower in EHL piglets, both at birth [27] and before weaning, compared to LW of the same age. Apparently, GR mRNA expression does not in accordance with the nuclear content of Sp1 protein in the liver. The role of Sp1 in the regulation of GR transcription is dependent on its binding to the GR promoters. In newborn piglets, Sp1 binding to GR 1–4 and 1–5 promoters did not differ between breeds, despite the fact that two Sp1 binding sites are predicted on the sequence of each 1–4 and 1–5 promoters and binding of Sp1 to both promoters are detected by ChIP analysis in our previous study [27]. To our surprise, however, we detected significantly higher Sp1 binding to GR promoter 1–9/10 in EHL piglets at preweaning stage in this study, which is associated with higher enrichment of acetylated histone 3 and enhanced expression of GR 1–9/10 mRNA variant. Histone acetylation results in relaxed chromatin structure and increases the recruitment of transcription factors and RNA polymerase II to initiate transcription [36], [37]. Therefore, it is reasonable that higher expression of GR exon 1–9/10 mRNA detected in EHL piglets is accompanied by increased enrichment of acetylated histone H3 on the promoter. However, it is curious why Sp1 binding to GR promoters in piglet liver differ so profoundly between two developmental stages.

One possible explanation on the developmental alteration in Sp1 binding might be the nature of GR promoters. The differentially expressed mRNA variants were GR 1–4 and 1–5 in newborn piglets, while in preweaning piglets, GR 1–9/10 was differentially expressed. GR promoter 1–9/10 is rich in GC content which is generally associated with constitutively expressed genes [38]. Sp1 is known to regulate the constitutive transcription of GR in most tissues [16], [20] and indeed GR 1–9/10 mRNA is detected as the predominant constitutive variant expressed in the liver of preweaning piglets. However, how the developmental transition of breed-dependent hepatic GR transcription is achieved remains unclear. Recently, it has been shown that the role switching of Sp1 during mouse liver development is dependent on the changes of the core promoter recognition and coactivator complex required for Sp1 activation [39].

p53 has been reported to cooperate with Sp1 to regulate the expression of various genes [40], [41]. For instance, p53 interacts with Sp1 to mediate transcriptional activation of the human BAX promoter [42]. Nevertheless, no direct evidences are available to indicate the role of p53 in Sp1 coactivator complex involved in GR transcriptional regulation. We observed significant positive correlation between p53 and GR mRNAs expressed in the liver of preweaning piglets, which supports the previous implications on possible associations between p53 and GR [23]–[25]. We further demonstrated the physical protein-protein interaction between p53 and Sp1 in nuclear lysates of porcine liver by using co-immunoprecipitation combined with Western blot analysis. Moreover, it is interesting that p53 and Sp1 interaction was breed-dependent; higher p53 and Sp1 binding was associated with higher Sp1 binding to GR 1–9/10 promoter in the liver of EHL piglets. Although p53 binding to GR 1–9/10 promoter did not differ between breeds, p53 may modulate GR transcription by its interaction with Sp1. Similar phenomena was reported previously in which p53 contributes to the positive regulation of GADD45 gene promoter by protein-protein interaction [43].

We further confirmed the role of p53 in the transactivation of porcine GR 1–9/10 promoter in a luciferase reporter gene assay in HepG2 cells. Doxorubicin, a well- known activator of the p53 pathway [44], [45], significantly increased p53 protein content in HepG2 cell lysates, as reported previously in other cell types [46], [47]. The Dox-induced activation of p53 pathway was associated with significantly increased transcriptional activity of porcine GR 1–9/10 promoter and higher content of endogenous GR protein in HepG2 cells. However, both basal activity and p53-mediated transactivation of porcine GR 1–9/10 promoter appear to be Sp1-dependent, because Mit significantly inhibited the basal activity of GR promoter 1–9/10 and blocked Dox-induced activation of GR promoter 1–9/10. These results further support our hypothesis that p53 cooperates with Sp1 to regulate breed-dependent expression of GR 1–9/10 mRNA in the liver of preweaning piglets.

In conclusion, we provide the first evidence that GR is expressed in a breed-specific manner in porcine liver. Higher hepatic GR expression in EHL piglets at preweaning stage attributes mainly to the enhanced transcription of GR mRNA variant 1–9/10, which is achieved from breed-specific interaction of p53 and Sp1 on porcine GR 1–9/10 promoter. Our findings may shed light on targeted GR regulation in order to improve the productive performances of pigs. However, further in-depth studies are needed to elucidate the complex molecular mechanisms underlying the temporal-, spatial and breed-dependent regulation of GR expression which involve interplay between various transcription factors/cofactors and the chromatin remodeling machinery, such as DNA and histone modifications, on GR promoters.

## Materials and Methods

### Ethics statement

The Animal Ethics Committee at Nanjing Agricultural University reviewed the protocol and approved this study specifically, with the project number 2009ZX08009-138B. The slaughter and sampling procedures strictly followed the “Guidelines on Ethical Treatment of Experimental Animals” (2006) No. 398 set by the Ministry of Science and Technology, China and the Regulation regarding the Management and Treatment of Experimental Animals”(2008) No.45 set by the Jiangsu Provincial People's Government. All of the LW and EHL piglets were sacrificed by electrical stunning followed by exsanguination prior to the study. The piglets were purchased from the respective pig breeding farms and a professional butcher was hired to help sacrifice the piglets.

### Animals

Preweaning piglets at the age of 25 days (d) were obtained from two neighboring pig breeding farms in Changzhou, Jiangsu Province, China. Five male piglets of EHL and LW breeds each were weighed and killed for sampling. Serum samples were prepared and stored at −20°C, and liver samples were taken within 5 min postmortem, snap-frozen in liquid nitrogen and stored at −80°C until further analysis.

### Radioimmunoassay for serum hormone levels

Serum cortisol concentration was detected with radioimmunoassay (RIA) using commercial kits from Beijing North Institute of Biological Technology (China). The detection limit for cortisol was 2 ng/mL. The inter- and intra-assay coefficients of variation were 10 and 15%, respectively.

### Western blot analysis of the nuclear content of transcription factors

Nuclear protein extracts were prepared from liver samples as previously described [48]. Briefly, about 200 mg frozen liver tissue were homogenized in 2 mL of low-salt buffer containing complete protease inhibitor, and incubated for 10 min on ice. After centrifugation for 1 min at 12 000 rpm at 4°C, the supernatant was retained as the cytosolic fraction, and the remaining pellet was dissolved with 200 µL high-salt buffer. Samples were kept on ice for 30 min, then centrifuged for 10 min at 12 000 rpm at 4°C. Supernatants were taken as nuclear fractions, protein concentrations were determined with a Pierce BCA Protein Assay kit.

Antibodies against GR (sc-1004, Santa Cruz), Sp1 (sc-14027, Santa Cruz), p53 (sc-126, Santa Cruz) and histone H1 (BS1656, Bioworld Technology) were used for Western blot analysis. A horseradish peroxidase-conjugated anti-rabbit or anti-mouse secondary antibody and chemiluminescence substrates (ECL; Amersham Bioscience AB) were used to detect the immuno-labeled bands. Images were captured using VersaDoc 4000MP system (Bio-Rad), and the band densities were calculated using Quantity-One software (Bio-Rad). Histone H1 was used as loading control in Western blotting analyses.

### RNA extraction and quantification of GR exon 1–9/10 mRNA variant

Total RNA was isolated from liver tissue using TRNzol Total RNA Kit (Tiangen Biotech Co. Ltd, Beijing) and was subsequently purified with the RNase-Free DNase Set (Promega, WI, USA), according to the manufacturer's instructions. The cDNA Synthesis Kit (Promega, Madison, WI, USA) was used to synthesize cDNA from 2 μg of total RNA according to the instruction of the manufacturer. Two microliters of diluted cDNA (1∶50) were used in real-time PCR using SYBR Green Real-time PCR Master Mix (TaKaRa, Japan) in Mx3000P (Stratagene, USA). We measured the relative expression of various proximal GR exon 1 mRNA variants and the total GR mRNA in liver of LW and EHL piglets using real-time quantitative PCR and the 2^−Δ ΔCt^ method [49]. The sequences of transcript-specific primer pairs were shown in Table 2. In order to determine the percentage of GR 1–9/10 mRNA variant relative to total GR mRNA, absolute quantitative measurement of exon 1–9/10 and total GR mRNA was performed. The standard curves were generated by amplifying a serial dilution of plasmid DNAs containing cDNA fragments of GR 1–9/10 and total GR, respectively.

**Table 2:** Nucleotide sequences of specific primers used in the study.

Target sites	Primers (from 5′ to 3′)	Application
GR 1–9/10	F: CCTGCTTTCACACGCTAA	Real-time PCR
	R: ACGCTGCTGGGGATTTC	
Total GR	F: CCAAGGAATCGCTGACCC	
	R: ATTGCTTCCTGAGCCTTTTG	
p53	F: ACCACCATCCACTACAACTTCA	
	R: CAGGACAGGCACAAACACG	
18S	F: TCGGAACTACGACGGTATCT	
	R: CGGAACTGAGGCCATGATTA	
GR promoter 1–9/10	F: CTCGGTCCCGCTTGCTCG	ChIP assay
	R: CGGCTCCTTCCACCCACGA	
GR promoter	F: CGGGGTACCAGGTCGGCCGCCGCCGC	Luciferase reporter assay
	R: CCGCTCGAGAATGGAGGAGGAGGAGGAGGA-3′	

Underlined sequences are digestion sites for the restriction enzymes Kpn I and Xho I.

### Co-immunoprecipitation assay

Co-immunoprecipitation was performed as previously described with minor modifications [50]. Nuclear protein were precleared using 60 μL of Protein A/G agarose beads (sc-2003, Santa Cruz) and incubated with 3 μg p53 antibody with rotation for 12 h at 4°C followed by a brief centrifugation, after addition of 60 μL of protein A/G-agarose bead suspension, the mixture was further incubated with rotation for 4 h at 4°C. After 3 washes with extraction buffer, the immunoprecipitated proteins were dissolved in 20 μL of laemmli buffer, boiled for 5 min at 95°C, and analyzed on 9% SDS-PAGE and transferred to nitrocellulose membrane for Western blot analysis of Sp1 protein and vice versa.

### ChIP assay for the analysis of Sp1 and H3Ac binding to GR promoter 1–9/10

ChIP assay was performed following previously described procedure [27], [51]. In brief, approximately 200 mg liver tissue was ground and fixed with formaldehyde. Then the pellet was resuspended in ice-cold hypotonic buffer, and allowed to swell on ice for 10 min, dounce homogenized and centrifuged. The pellet was washed once with cold PBS and resuspended in SDS lysis buffer. Cross-linked chromatin was sheared by sonication to achieve an average DNA length of ∼500 bp. Two micrograms of antibodies against Sp1 (sc-14027, Santa Cruz), p53 (sc-126, Santa Cruz) and H3Ac (06–599, Millipore) were used respectively for ChIP analyses. Normal rabbit IgG (sc-2027, Santa Cruz) was used as negative control [52], [53]. Aliquots of chromatin (equivalent to 50 μg DNA) were incubated with respective primary antibody overnight at 4°C and precipitated with 60 μL protein A/G agarose beads for 2 h at 4°C with rotation. The antibody/chromatin complex was then washed and collected for subsequent reverse cross-linking. The DNA released from reverse cross-linking was then recovered by phenol/chloroform extraction and ethanol precipitation followed by resuspension in nuclease-free water for PCR amplification. The amount of precipitated DNA was calculated relative to the input, the results are presented as a percentage of the input DNA.

### Cell Culture

HepG2 cells were cultured at 37°C in a humidified atmosphere containing 5% CO_2_ and maintained in DMEM (Gibco Life Technologies) in tissue flasks supplemented with 10% heat-inactivated FBS (Hyclone), penicillin (100 units/mL), and streptomycin (0.1 mg/mL).

### Luciferase reporter gene assay

The 5′-flanking sequence of porcine GR exon 1–9/10 (promoter 1–9/10, −3149/−2724), which includes also exon 1–8, was amplified by PCR using the genomic DNA extracted from pig liver as template, using primers shown in Table 2. PCR product was inserted into a firefly luciferase expression vector, pGL3-Basic (Promega, Madison, WI), and the reporter plasmid construct was transfected to HepG2 cells using lipofectamine 2000 reagent (Invitrogen, USA) as previously described [54]. In brief, approximately 1×10^5^ cells/well were seeded in 24-well (for Dual Luciferase Reporter Assay) or 4×10^5^ cells/well in 6-well (for Western blot analysis of GR and p53 protein) plates (Corning) and cultured for 24 hours, each well was transfected with 300 ng of reporter construct and 30 ng of pRL-TK plasmid which was used to monitor transfection efficiency. 24 hours post-transfection, cells were treated with 10 nM and 100 nM Dox, a p53 activator [55], for 12 hours. In another experiment, cells were treated with 100 nm of Dox or Mit or the combination of both, for 12 hours. Subsequently, cells were harvested, and the luciferase activity was measured by a luminometer (Glomax, Promega) via Dual Luciferase Reporter Assay System (Promega) according to the manufacturer's instruction. For Western blot analysis of GR and p53 protein, cells were treated with 100 nM Dox for 12 hours, then washed with PBS and lysed in RIPA buffer (Biyuntian Ltd, Nantong, China). Dox was purchased from Tocris Cookson Ltd. (Avonmouth, UK). Sp1 inhibitor Mit was purchased from Sigma, USA.

### Statistical analysis

All data were presented as mean ± SEM, and were analyzed using Independent-Samples T Test with SPSS 13.0 for Windows (SPSS, Inc., Chicago, IL). Statistical significance was set at *P*<0.05. Pearson's correlation coefficient was used to test the correlation between p53 and GR mRNA in liver of preweaning piglets.

## Supporting Information

Figure S1 **Standard curves used in the absolute qPCR for determining copy numbers of GR 1–9/10 mRNA and total GR mRNA in liver of preweaning piglets.** The standard curves in the absolute qPCR were generated by amplifying a serial dilution of the plasmid DNAs containing cDNA fragments of GR 1–9/10 (A) and total GR (B), respectively.
(TIF)
