# Supplementary figures and images for: p53 Cooperates with Sp1 to Regulate Breed-Dependent Expression of Glucocorticoid Receptor in the Liver of Preweaning Piglets
Source: PLoS One. 2013 Aug 7;8(8):e70494. doi: 10.1371/journal.pone.0070494 (PMC3737268; doi:10.1371/journal.pone.0070494)

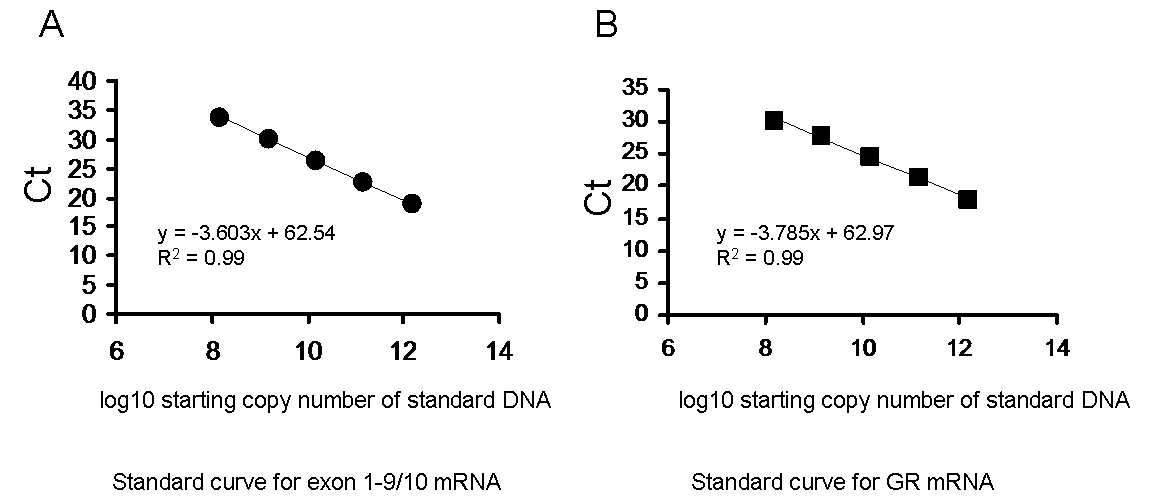

Supplement: Figure S1 — Standard curves used in the absolute qPCR for determining copy numbers of GR 1–9/10 mRNA and total GR mRNA in liver of preweaning piglets. The standard curves in the absolute qPCR were generated by amplifying a serial dilution of the plasmid DNAs containing cDNA fragments of GR 1–9/10 (A) and total GR (B), respectively. (TIF) [file pone.0070494.s001.tif]
